# Ubiquitin Specific Protease 26 (USP26) Expression Analysis in Human Testicular and Extragonadal Tissues Indicates Diverse Action of USP26 in Cell Differentiation and Tumorigenesis

**DOI:** 10.1371/journal.pone.0098638

**Published:** 2014-06-12

**Authors:** Matthew S. Wosnitzer, Anna Mielnik, Ali Dabaja, Brian Robinson, Peter N. Schlegel, Darius A. Paduch

**Affiliations:** 1 Department of Urology, Weill Cornell Medical College, New York, New York, United States of America; 2 Department of Pathology, Weill Cornell Medical College, New York, New York, United States of America; Cardiff University, United Kingdom

## Abstract

Ubiquitin specific protease 26 (USP26), a deubiquitinating enzyme, is highly expressed early during murine spermatogenesis, in round spermatids, and at the blood-testis barrier. USP26 has also been recognized as a regulator of androgen receptor (AR) hormone-induced action involved in spermatogenesis and steroid production in *in vitro* studies. Prior mutation screening of USP26 demonstrated an association with human male infertility and low testosterone production, but protein localization and expression in the human testis has not been characterized previously. USP26 expression analysis of mRNA and protein was completed using murine and human testis tissue and human tissue arrays. USP26 and AR mRNA levels in human testis were quantitated using multiplex qRT-PCR. Immunofluorescence colocalization studies were performed with formalin-fixed/paraffin-embedded and frozen tissues using primary and secondary antibodies to detect USP26 and AR protein expression. Human microarray dot blots were used to identify protein expression in extra-gonadal tissues. For the first time, expression of USP26 and colocalization of USP26 with androgen receptor in human testis has been confirmed predominantly in Leydig cell nuclei, with less in Leydig cell cytoplasm, spermatogonia, primary spermatocytes, round spermatids, and Sertoli cells. USP26 likely affects regulatory proteins of early spermatogenesis, including androgen receptor with additional activity in round spermatids. This X-linked gene is not testis-specific, with USP26 mRNA and protein expression identified in multiple other human organ tissues (benign and malignant) including androgen-dependent tissues such as breast (myoepithelial cells and secretory luminal cells) and thyroid tissue (follicular cells). USP26/AR expression and interaction in spermatogenesis and androgen-dependent cancer warrants additional study and may prove useful in diagnosis and management of male infertility.

## Introduction

Infertility affects up to 15% of couples, and it is estimated that approximately 50% of cases involve some degree of male factor infertility. Male factor infertility may be attributed to genetic defects in up to 30% of cases. Wang et al. initially identified X-linked genes expressed exclusively in the testis, and USP26 has been previously considered to be testis-specific[1]. Ubiquitin specific protease 26 (USP26) is an X-linked gene located at Xq26.2 with a single exon encoding a 93 kDa protein. USP26 is part of a larger family of deubiquitinating enzymes (DUBs), which have high levels of substrate specificity expression [2]-[4]. Ubiquitination regulates cell proliferation and function through proteasome 26 and other systems [5]. Deubiquitination, an opposing process, can prevent structural and regulatory proteins from undergoing degradation. The delicate balance between ubiquitination and deubiquitination is essential for cellular function. Each phase of mammalian spermatogenesis necessitates different activities of the ubiquitin system[6], . Adult murine models indicate that USP26 is highly expressed in spermatogonia types A and B, pre-leptotene spermatocytes, round spermatids, and at the blood-testis barrier[8]. USP26 protein localization and expression in the human testis, however, has not been well characterized[8]–[13].

Our group and others have described specific mutations in USP26 which are associated with male infertility; however the mechanism of how such mutations modulate testicular function is unknown [10], [12], [14]–[17]. In one of the most comprehensive single nucleotide polymorphism (SNP) studies based on a genome wide association study and published male infertility genes, 147 SNPs were evaluated and 14 had significant association with male infertility including USP26 (rs35397110)[18].

Based on mutational screening-phenotype analysis we hypothesize that USP26 plays a critical role in the regulation of early stages of maturation including mitotic divisions of spermatogonia or migration of primordial germ cells, as mutations in USP26 occur in patients with Sertoli cell only syndrome and early maturation arrest[10], [12]. USP26 may also be involved with regulation of protein turnover (such as histone removal) prior to meiosis as well as the movement of germ cells across the blood-testis barrier.

Recent discovery that USP26 regulates androgen receptor (AR) transcriptional activity provides additional support for the role of USP26 in spermatogenesis, Leydig cell steroidogenesis and Sertoli cell factor secretion[19]–[21]. The binding of testosterone or its metabolite 5α-dihydroxytestosterone (DHT) to AR induces loss of heat shock proteins, receptor dimerization, promoting AR to bind to its response elements in the nucleus and to recruit coregulators to promote target gene expression. Androgen receptor coregulators are recruited by AR and assure the ability of AR to influence gene expression by modulating AR folding, AR stability, and subcellular localization[22]. A loss-of-function screen in AR signaling was conducted by Dricac et al., which identified USP26 as a novel regulator of hormone-induced AR signaling, binding nuclear AR and reversing AR ubiquitination (the result of hormonal stimulation). USP26 inhibition in the presence of androgen stimulation consistently represses hormone induced-AR target gene expression.

Disrupted regulation of AR signaling has been described in the development and progression of multiple cancers including testicular cancer, breast, and thyroid cancer. Given USP26 interaction with the androgen receptor, we conducted this study to characterize USP26 as a potential target affecting human testicular function and fertility and androgen-dependent cancers.

## Methods

### Ethics Statement

This research study including human and animal tissue, has been approved by Weill Cornell Medical College IRB (IRB 1209013045 and IRB 1202012193 and IRB 0102004794) in accordance with principles of the Declaration of Helsinki. Written informed consent from the donor or next of kin was obtained for use of human tissues samples for research when possible. If patients were lost to follow-up, the Weill Cornell Medical College IRB waived the need for consent. Tissue samples were collected in accordance with Weill Cornell Medical College Department of Pathology (http://www.cornellpathology.org) protocols approved by the IRB. All animal tissue procedures were approved by the Weill Cornell Medical College IRB according to the Cornell Institutional Animal Care and Use Committee (IACUC). Mice were cared for according to Cornell IACUC principles and the Guiding Principles for Biomedical Research Involving Animals.

### Identification of antibody for USP26/Cloning vectors

**Figure 1:**
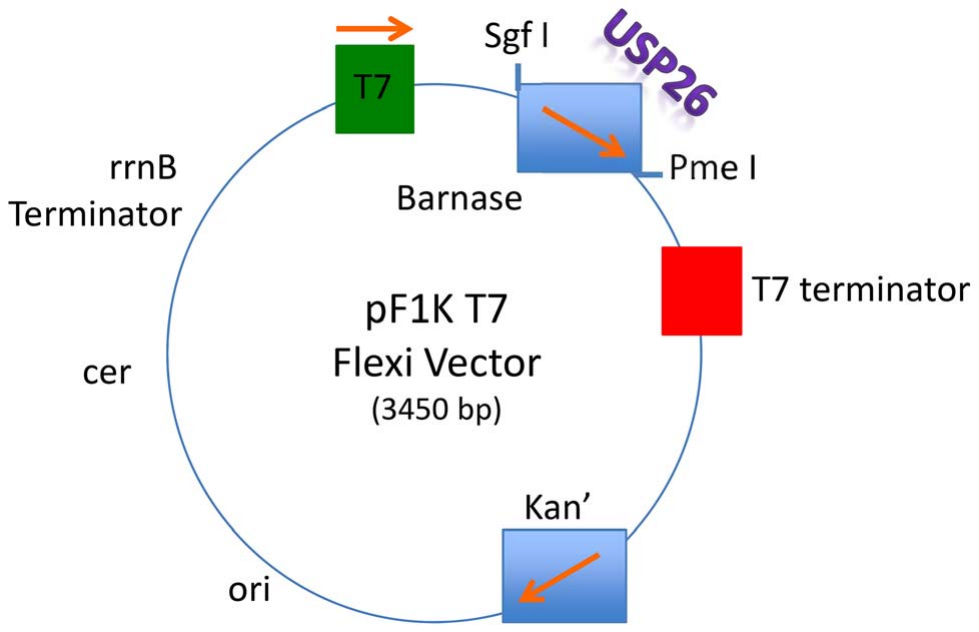
USP26 pF1K and pFN2A GST Flexi Vectors. Adapted from pF1KT7 Flexi Vector map with sequence reference points (Promega: Madison, WI)

To test specificity of commercial anti-USP26 antibody, the USP-26-GST vector was first constructed and then expressed by our group for further analysis using western blot. Wild type USP26 has been subcloned into pF1K and pFN2A GST Flexi vectors, sequenced and co-expressed in BL21 (DEC3) *E. coli* together with a classic substrate for deubiquitinating enzymes (Ub-β-galactosidase) (Figure 1) [23], [24]. Conjugated Ub-β-galactosidase plasmid (provided by Dr. Hochstrasser, Yale University- original constructs of pACUb-R-β-gal and pACUb-M-β-gal are based on the pACYC184 plasmid) was linearized using Ahd I and subsequently cDNA of Ub-R-β-gal and Ub-M-β-gal was formulated using PCR and SgfI and PmeI site-specific overhanging primers. The human cDNA was subcloned into described vectors and transformed into BL21(DE3) *E. coli* strain. The original vectors were subcloned in plasmid for use in deubiquitination assays and immunodetection. The transformant was grown on kanamycin selected LB plates with X-gal. Those colonies were selected and the presence of constructs verified by sequencing.

### Real-Time PCR

#### Evaluation of mRNA USP26 expression using quantitative PCR

Quantitative PCR was performed using cDNA synthesized from total RNA obtained from benign and malignant frozen tissue from normal testis (2 healthy males with normal spermatogenesis), thyroid cancer (3 males, and 2 females with papillary thyroid carcinoma), and breast cancer (5 females with ductal breast carcinoma). Frozen tissue identified by a single pathologist was weighed, thawed, and homogenized. Total RNA was obtained using Exiqon miRCURY RNA Extraction Kit (Roche Diagnostics, Exiqon). RNA concentration was measured via Qubit fluorometer (Life Technologies) and purity and RNA integrity were confirmed (Agilent Bioanalyzer). cDNA was synthesized from 1 µg of purified total RNA using the Transcriptor First Strand cDNA Synthesis Kit (Roche Diagnostics, Indiana) with random hexamer primers. cDNA was subsequently stored at -20 degrees Celsius until utilized. USP26 and AR mRNA levels were measured using dual-color multiplex qRT-PCR with the Universal Probe Library (UPL) hydrolysis probe set on Roche LightCycler 480 instrument (Roche Diagnostics, Indiana). TATA-binding protein (TBP) served as the housekeeping gene for relative quantification experiments due to consistent and reliable TBP expression in testis, breast, and thyroid samples. TBP has been recognized to be an ideal housekeeping gene for thyroid studies[25].

Multiplex assays were designed with the UPL Assay Design resource (http://www.roche-applied-science.com/webapp/wcs/stores/servlet/CategoryDisplay?catalogId=10001&tab=&identifier=Universal+Probe+Library&langId=-1&storeId=15016#tab-0). Universal Probe Library permits optimal design of real time PCR experiments with increased specificity. USP26 was detected with the forward and reverse primers (0.8 µM) L-CGATGATATGCGGGTGTTAG and R-GTACCCAGTGCAACGCCTAT with UPL probe #79. AR was detected with the forward and reverse primers (0.6 µM) L-GCCTTGCTCTCTAGCCTCAA and R-GTCGTCCACGTGTAAGTTGC with UPL probe#14. TBP mRNA was detected using Human TBP Gene assay (Roche Diagnostics).

All qRT-PCR reactions incorporated identical total RNA quantity, and all reactions were performed in duplicate using 96-well plates. Each 20 ul reaction mixture contained 1x Lightcycler 480 Probes Master Mix (Roche Diagnostics), 1 µL cDNA, UPL probe (200 nmol/l), TBP probe (100 nmol/l), and forward and reverse primers for USP26#79 and TBP or AR#14 and TBP (Roche Diagnostics). Each run included denaturation at 95°C for 5 minutes, followed by 45 cycles of 95°C for 10 seconds, and 60oC for 30 seconds, followed by a cooling cycle to 55oC. Fluorescence versus cycle number curves for each sample were converted to log-linear curves, and crossing points were analyzed using the second derivative maximum method of Roche LightCycler software. Amplification of the specified fragment was confirmed by gel electrophoresis. RNA expression was normalized to expression of the housekeeping gene TBP. USP26/TBP and AR/TBP expression ratio was calculated using LightCycler 480 software (Roche Diagnostics) for relative quantification. Standard curves were generated (via triplicate multiplex RT PCR reaction) for each target (USP26, AR) and reference (TBP) gene using serial 5-fold dilutions of cDNA from a patient with normal spermatogenesis for PCR efficiency corrections in all relative quantification analysis. Software application of PCR efficiency corrections were based on the standard curves for USP26 (1.8), AR (1.81), and TBP (1.7). Since USP26 is intron-less, every amplification run was performed with negative control (no reverse transcriptase) to exclude genomic amplification.

### Immunofluorescence (IF)

Formalin-fixed/paraffin-embedded and frozen normal testis tissue as well as formalin-fixed/paraffin-embedded benign/malignant breast and thyroid tissue were utilized for analysis. Multi-channel IF with antibodies against USP26 (rabbit polyclonal IgG, Ab101650, Abcam) and AR (mouse monoclonal IgG, AR441: sc-7305, Santa Cruz Biotechnology) to define expression patterns of USP26 very early during spermatogenesis and during early meiotic divisions. Formalin-fixed/paraffin-embedded slides were treated with xylene, ethanol, rehydrated with 1xPBS, and permeabilized with PBT (0.2% Triton X-100). Frozen tissue slides were fixed in 4% paraformaldehyde for 10 minutes, followed by rehydration and permeabilization in PBT. Slides were subsequently blocked using normal goat serum for 1.5 hours. After application of primary antibody (20 ug/mL for USP26 and 4 ug/mL for AR), slides were incubated at 4 degrees Celsius overnight. After rinsing with PBT, secondary antibodies Alexa Fluor 488 (goat anti-rabbit IgG, A-11008, Life Technologies) and Alexa Fluor 555 (goat anti-mouse IgG, A21422) were applied at 1∶1000 concentration for 1 hour at 37°C. Following rinse with 1x PBS, ProLong Gold AntiFade with DAPI (P36931, Life Technologies) was used for nuclear staining. Images were acquired using Nikon E800 epifluorescent microscope with a deconvolution mechanism controlled by Vincent Associates shutter system, and analyzed with Image Pro-Plus 4.5 software. The USP26 emitted green fluorescence, which contrasted well with red fluorescence of AR and blue fluorescence of the nucleus. The software was initially optimized and the analysis algorithm subsequently applied to testis tissue[26]–[28].

### Immunohistochemistry

Testicular tissue samples (fixed in Bouin's solution, and prepared in 5 µm sections) were obtained from patients with SCO. USP26 was identified using C-terminal antibody, rabbit anti-human anti USP26 C- terminal (region 916–931) (Abgent, AP2151b) primary antibody (dilution 1∶50) to detect USP26. This was followed by secondary antibody conjugated to Streptavidin–horseradish peroxidase (Histostain-SP DAB broad kit, Zymed 85–9643). Antigen exposure was completed using citric-buffer and microwave protocol. C-terminal antibody was localized using secondary alkaline phosphatase (AP)-conjugated antibody NBT/BCIP (Pierce) was used to detect activity of AP (purple-brown color). Human muscle slides were used as a negative control.

### Microarray Dot Blot Protein Expression

Protein array was utilized to screen multiple normal and neoplastic human tissues for USP26 expression levels. Three human tissue extract microarrays derived from cancer tissue from various organs were analyzed. The blot was blocked and incubated with primary antibody and washed with TBS/Triton wash. Secondary antibody coupled with HRP were used to detect anti-USP26 antibody. ECL was utilized for chemiluminescence detection. ImageJ (NIH) software was used after background/linearity correction. Expression ratios were calculated as cancer/normal tissue normalized optical density. Expression was confirmed using protein lysates.

Statistical analysis was performed using JMP software (SAS, 2012). Serum hormonal parameters, average testicular volume, age, and mean USP26/TBP and AR/TBP ratios were analyzed in relation to microdissection TESE outcome with Mann-Whitney test and linear regression. Performance of USP26/TBP and AR/TBP expression ratio in predicting retrievable sperm from microdissection TESE was determined by logistic regression and receiver operating characteristic (ROC) curve analysis.

## Results

### Antibody Evaluation

Given lack of adequate positive and negative controls to test different antibodies to study human samples, we cloned and expressed human USP26-GST and subsequently verified specificity of various commercial antibodies. Wild type USP26, which has been subcloned into pF1K and pFN2A GST Flexi vectors, sequenced and expressed in BL21 (DEC3) *E. coli*. USP26 was co-expressed in *E. coli* together with a vector carrying classic substrate for deubiquitinating enzymes (Ub-β-galactosidase). The insert was confirmed by sequencing selected vectors. Anti-GST and commercial anti-USP26 antibodies were used to select the optimal antibody using Western blots.

The best antibody to identify native human USP26 protein from testis for Western blot is N-terminal, polyclonal antibody (Abgent) detecting 100 kDa product with minimal background. Western blot illustrating antibody against N-terminal was used to detect USP26 in HeLa cells, testis tissue for men with normal spermatogenesis, human bladder, and human ureter (results not shown). Although the N-terminal antibody is best for western blot, the C-terminal antibody has been determined to be specific for USP26 in IHC applications. The N-terminal antibody was optimal for IF applications given our slide preparation protocol.

### USP26 and AR mRNA expression by qRT-PCR in testis and non-testis tissues

**Figure 2:**
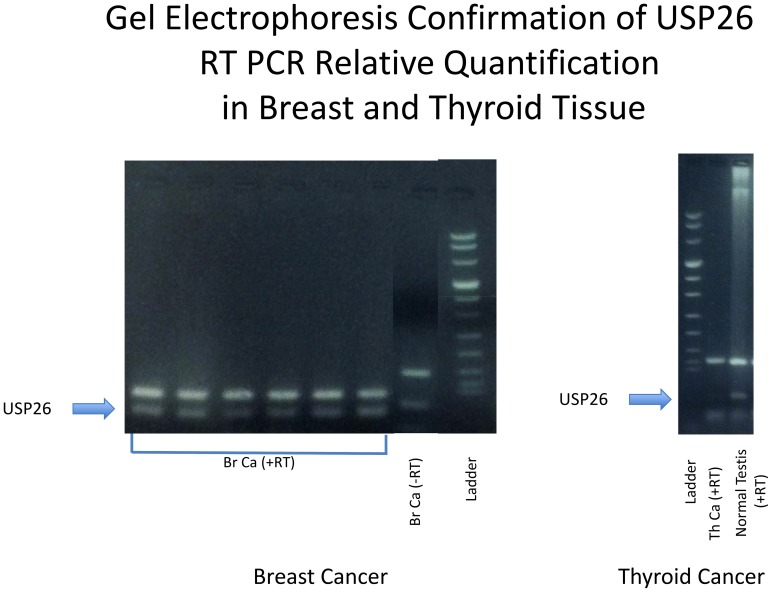
Gel Electrophoresis demonstrating USP26 mRNA expression in thyroid and breast cancer tissue following qRT-PCR. USP26 mRNA expression in breast and thyroid cancer confirmed by gel electrophoresis with amplicon for USP26 noted in breast cancer, thyroid cancer, and normal testis.

USP26 expression was verified by quantitative real time-PCR (qRT-PCR) in human normal testis tissue. Specifically, normal human testis tissue demonstrates expression of USP26 between 20 and 1000 fold of the expression in thyroid cancer and at least 3 fold greater than breast cancer tissue. This represents the first demonstration of USP26 in organs other than testis, altering the previous assumption that USP26 expression was testis-specific (Tables 1A and 1B). Real-time PCR results were verified by gel electrophoresis (Figure 2). No expression of USP26 was identified in human brain, or murine epididymis, kidney, liver establishing reliable negative controls for expression studies.

**Table 1:** USP26 and Androgen (AR) mRNA Expression Levels in Testis, Breast and Thyroid Tissue.

Sample	Histology	Mean Cp Target	Mean Cp Reference (TBP)	Target/Reference (TBP)
		***USP26***		
Th2T	Thyroid (cancer)	38.5	28.5	0.901
Th5B	Thyroid (benign)	0	29.9	0
Th5T	Thyroid (cancer)	37.6	28.5	1.78
T436	Normal testis	31.0	25.5	44.0
		***AR***		
Th2T	Thyroid (cancer)	28.9	27.8	0.169
Th5B	Thyroid (benign)	27.8	27.9	0.169
Th5T	Thyroid (cancer)	24.2	26.3	0.639
T436	Normal testis	27.9	25.4	.0372
				
		***USP26***		
Br9T	Breast (cancer)	34.2	29.1	31.82
T436	Normal testis	30.4	25.9	87.02
				
		***AR***		
Br9T	Breast (cancer)	25.9	28.6	0.913
T436	Normal testis	28.1	26.3	.0538

Additionally we analyzed androgen receptor (AR) mRNA expression simultaneously with USP26 and noted that AR expression was lower in testis tissue than breast or thyroid tissue with AR target/reference expression ratios for normal testis ranging between .0372 to .0915, thyroid (benign) ranging between .169 to .313, thyroid (malignant) between .169 to 1.494, breast (benign) between .907 to 1.703, and breast (malignant) between .525 to 1.020 (Table 1 and additional data not shown). Breast cancer exhibited lower AR expression than benign breast tissue. Expression levels of AR varied in thyroid tissue without clear differences in expression between benign and malignant tissue.

### Localization of human USP26 protein

**Figure 3:**
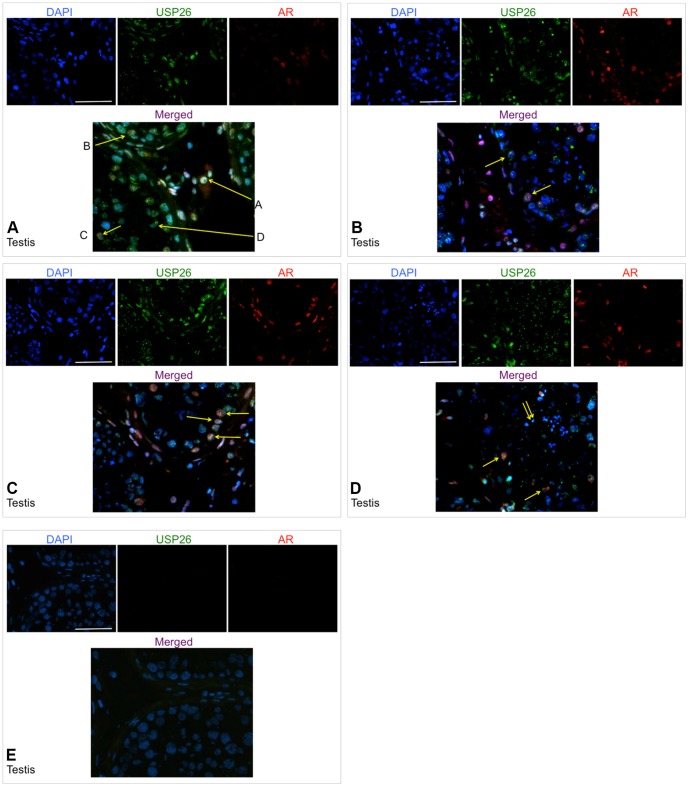
USP26 and AR protein expression in normal testis by immunofluorescence. All images of stained sections were captured at 400x total magnification; scale bar = 100 µm. **3A**: Colocalization of USP26 and AR in nucleus and cytoplasm of Leydig cell (A), spermatogonia (B), primary spermatocyte (C), and Sertoli cell (D) in normal human testis (frozen tissue). **3B**: Partial colocalization of USP26 and AR in nucleus and cytoplasm of Sertoli cell (left arrow) and spermatogonia (right arrow) of normal human testis (frozen tissue). **3C**: Colocalization of USP26 and AR in nucleus and cytoplasm of Sertoli cells (left arrow) and spermatogonia (right arrows) of normal human testis (frozen tissue). **3D**: USP26 expression in spermatids (double arrows), and USP26/AR partial colocalization in early cells of spermatogenesis (single arrows) of normal human testis (frozen tissue). **3E**: Negative control demonstrating specificity of antibody without staining of USP26 or AR, or auto-fluorescence of normal human testis (frozen).

**Figure 4:**
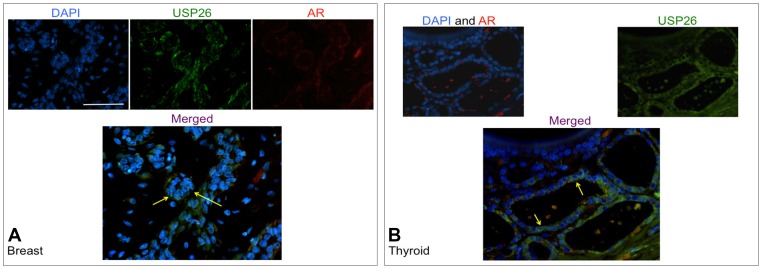
USP26 and AR protein expression in breast and thyroid tissue by immunofluorescence in formalin-fixed, paraffin-embedded human tissue. **4A**: USP26 staining in benign breast tissue (paraffin-embedded fixed), mostly cytoplasmic with some nuclear staining in secretory luminal cells (right arrow) and myoepithelial cells (left arrow). **4B**: USP26 staining in benign thyroid follicular cells (arrows), mostly cytoplasmic localization.

Immunofluorescence studies were conducted using formalin-fixed/paraffin-embedded and frozen normal testis, and formalin-fixed/paraffin-embedded benign and malignant breast and thyroid tissue (5 um thickness). Results in five human patients with normal testis histology demonstrated consistent USP26 expression in the nucleus and less in the cytoplasm of Leydig cells with significant colocalization with androgen receptor in the nucleus (Figure 3A). Additionally, select spermatogonia, primary spermatocytes, and Sertoli cells demonstrate nuclear USP26 expression with partial colocalization with AR (Figure 3B, 3C). Further, round spermatids are noted to have USP26 expression (Figure 3D). Negative control is shown without USP26/AR expression establishing staining specificity (Figure 3E). The differentiation between Sertoli cell and spermatogonial nuclei was based on morphologic appearance and histologic analysis. Sertoli cells typically have smaller and more slender, elongated ovoid, pear-shaped nuclei compared with spermatogonia which have larger and rounder nuclei. Sertoli cells have indented nuclear envelopes and large nucleoli which are distinctly different than spermatogonia which have less defined nuclei and nucleoli away from the nuclear envelope. Additionally, Sertoli cell nuclei often appear 1 cell away from basement membrane, while spermatogonia nuclei sit on the basement membrane. Histologically the difference between Sertoli cells and spermatogonia was confirmed by vimentin staining (to identify Sertoli cells) and DDX4 (VASA) staining (to stain germ cells)[29]. Breast tissue from 5 patients also demonstrated cytoplasmic and nuclear USP26 expression in myoepithelial cells and secretory luminal cells of benign tissue (Figure 4A). Thyroid tissue from 5 patients exhibited cytoplasmic and perinuclear USP26 staining in follicular cells of benign tissue which co-localized with AR in specific regions (Figure 4B).

**Figure 5:**
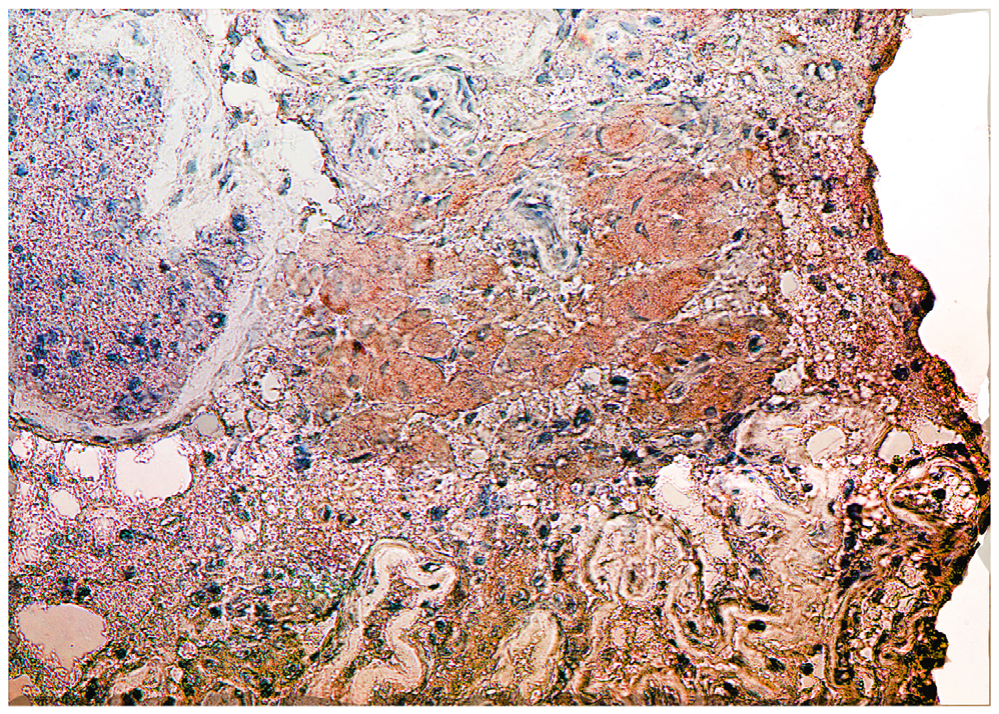
Localization of human USP26 protein. In this patient with Leydig cell hyperplasia and a lack of germ cells in tubules (SCO), USP26 was identified using C-terminal antibody in human Leydig cells, with signal detected solely in Leydig cells. These staining pattern findings were validated in a total of four patients, all with similar findings.

Within human testis, USP26 was expressed in Leydig cells and early spermatogonia, as expected from our previously published mutational analysis showing USP26 mutations associated with infertility and low testosterone in men (Figures 3 and 4) and our murine studies (not shown). In biopsies of human testicular tissue with Leydig cell hyperplasia and a lack of germ cells in tubules, USP26 was identified by IHC using C-terminal antibody with signal detected solely in Leydig cells in four human patients all with similar staining patterns (Figure 5).

### Protein array results

USP26 is expressed in testis as well as other organs, including prostate, ovary, cervix, and breast. Thyroid, adrenal, and stomach cancer highly overexpress USP26, while prostate, and ovarian cancers have lower expression of USP26. Breast cancer tissue may either overexpress or underexpress USP26. Protein array results are shown (Table 2). Only thyroid, adrenal, stomach, bladder, and liver cancer exceeded expression threshold of 3-fold difference. USP26 was especially overexpressed in thyroid cancer as compared to normal thyroid. The expression in breast cancer and in cervical cancer were lower than in normal tissues. This may be explained by fact that only hormone dependent breast cancer overexpressed USP26 in microarray experiments. Expression in cervix and cervical cancer is very highly consistent with high expression levels of USP26 in HeLa cells.

**Table 2:** Mean absolute expression of human USP26 protein in an array of human normal and neoplastic tissues.

Tissue	Normal	Cancer	Expression ratio	Cancer vs. normal
**Thyroid**	2	3193	1596.5	+
**Adrenal**	255	2721	10.7	+
**Stomach**	787	4618	5.9	+
**Bladder**	4139	16057	3.9	+
**Liver**	692	2156	3.1	+
**Kidney**	2943	7400	2.5	+
**Brain**	4581	10681	2.3	+
***Colon***	*4604*	*5302*	*1.2*	*-*
***Cervix***	*12383*	*10093*	*1.2*	*-*
***Uterus***	*6415*	*5968*	*1.1*	*-*
***Skin***	*12222*	*7501*	*1.6*	*-*
**Prostate**	5043	1931	2.6	-
**Ovary**	6542	1781	3.7	-
**Rectum**	3749	1013	3.7	-
**Esophagus**	1233	236	5.2	-
**Lung**	4974	784	6.3	-
**Breast**	6517	519	12.6	-

Table 2 demonstrates USP26 protein expression in non-testicular tissue including androgen-dependent cancers. Expression ratio is defined as higher expression divided by lower expression. Cancer versus normal column is directional change showing increased or decreased in cancer relative to normal tissue from each organ (+ =  increase as compared to normal tissue; − =  decrease in expression compared with normal tissue). Expression ratio then used to classify expression pattern (Green =  overexpression; White =  ratio +2 to −2 (equivocal expression), Red =  lower expression compared to normal tissue).

## Discussion

USP26 is a novel candidate gene for male infertility with USP26 expression previously reported in murine spermatogonia (types A and B), Sertoli cells, and potentially embryonic stem cells. This study provides detailed description of USP26 localization using multiple complementary detection techniques: IHC, IF, protein array, and quantitative analysis of mRNA expression. Our results demonstrate USP26 mRNA and protein expression in human testicular tissue, for the first time, occurring most prominently in Leydig cells. Additionally, selected spermatogonia and primary spermatocytes, round spermatids, and Sertoli cells demonstrate USP26 protein expression. Expression during early and late spermatogenesis may reflect meiotic sex chromosome inactivation (MSCI) characterized by transcriptional silencing of genes on both the X and Y chromosomes in mid to late pachytene spermatocytes with incomplete post-meiotic X repression allowing X chromosome reactivation after MSCI[30]–[32]. However, USP26 is not testis-specific, as it was previously characterized, with mRNA and protein expression identified in multiple other human organ tissues (benign and malignant) including breast (myoepithelial cells and secretory luminal cells) and thyroid tissue (follicular cells). For the first time, to our knowledge, colocalization with androgen receptor in human testis has been confirmed in Leydig cells, and early cells of spermatogenesis, as well as in follicular cells of benign and malignant thyroid tissue.

Since USP26 seems to be preferentially expressed in Leydig cells, spermatogonia, and primary spermatocytes, it is likely that USP26 affects regulatory proteins of early spermatogenesis, including androgen receptor[19]. Our mutation screening revealed that USP26 mutation is associated with infertility and low testosterone levels [10], [14], [15]. We hypothesize that hypogonadism is secondary and subsequent to aberrations in Leydig cell function and spermatogenesis. Our finding of USP26 expression predominantly in Leydig cells is intriguing. This confirms prior murine findings of USP26 expression in Leydig cell and spermatids for the first time in humans[32]. Indeed, impaired Leydig cell function and impaired spermatogenesis may represent a congenital dysfunction caused by a testicular dysgenesis or Leydig cell dysfunction during fetal or infant development[20], [33], [34]. In prior work by our group, sequence alterations in the USP26 gene were identified in approximately 11% of patients with severe oligospermia and non-obstructive azoospermia but were not identified in fertile controls. Patients with USP26 mutations had statistically significant lower testosterone levels (277 ng/dl vs. 405 ng/dl) as well as reduced testicular size (8.5 ml vs 12.4 ml). This is noteworthy since particular USP26 mutations were associated with impairment in testosterone possibly due to impaired USPS26-androgen receptor interaction in Leydig cells. Additionally, an association between impaired Leydig cell function and testicular cancer has been indicated by identification of increased LH levels and generally low testosterone levels in men with testicular carcinoma *in situ* (precursor cells of testicular cancer) when compared to men without CIS of the testis[35]. Abnormal semen parameters associated with testicular cancer have been well documented and supports further study of USP26 in testicular cancer models [36], [37].

Confirmation of colocalization of USP26 with AR by immunofluorescence in normal testicular tissue provides the first evidence of this interaction in humans. The AR signaling pathway is critical for male sexual differentiation and maturation, spermatogenesis maintenance, and prostate development and function. AR is known to be expressed in Sertoli, Leydig cells, peritubular myoid cells, and perivascular smooth muscle cells in the testis[38]. There is historical debate that has existed regarding AR expression in germ cells, but several studies describe that AR is present in germ cells in different species including humans[39]–[42], while other groups describe lack of AR expression more often in rodent studies[43]–[45]. Regulation of AR by various coregulators has been observed in testicular, prostate, bladder, breast, and thyroid cancer[29], [32], [45]. For example, multiple deubiquitinating enzymes have been identified which influence AR signaling in both cytoplasm and nucleus including TRIM68 which is specifically upregulated in prostate cancer and influences AR stability[46], [47]. Small-interfering RNA mediated knockdown of AR coactivators has resulted in marked decreases in androgen-induced bladder cancer cell proliferation[29], [48]. Transfection of GFP-USP26 into HEK293 cells revealed nuclear localization with an accumulation and colocalization with AR in subnuclear foci which is similar to the effect of a specific AR mutation of the DNA-binding domain which confers androgen insensitivity syndrome (AIS) in patients via impaired ligand-dependent nuclear translocation, subnuclear foci formation, and intranuclear mobility of the receptor[19], [49], [50]. The combined cytoplasmic and nuclear localization of USP26 is substantiated by localization prediction of USP26 using PSORT II software which demonstrates 6 pat4 and 2 pat7 nuclear localization signals (NLS) with Reihnardt's method predicting nuclear localization with reliability of 89 points. Therefore, it is logical that USP26 was localized to nuclei of Leydig cells and proliferating spermatogonia A and B, which corroborates a critical role in regulation of mitosis in spermatogonia.

With mRNA and protein expression of USP26 confirmed in breast and thyroid cancer, the interaction of USP26 and AR may play a significant role in tumorigenesis. With abnormal USP26 expression (both under and overexpression) identified in breast tissue, additional investigation is warranted given the known USP26 interaction with androgen receptor. Preclinical studies have demonstrated that androgens can induce proliferative changes in breast cancer cell lines and promote tumorigenesis in animal models. Preclinical studies have demonstrated that androgens can induce proliferative changes in breast cancer cell lines and promote tumorigenesis in animal models. Both estrogen and testosterone can induce tumorigenesis in young-adult female rats with testosterone acting as a tumor promoter in this model[51]. Large-scale retrospective reviews of AR status in breast cancer indicate a significant association between AR expression and overall survival, which remain significant even when controlled for ER status[52]. Higher AR expression was significantly correlated with a decreased risk of recurrence and death when compared with patients having breast cancer with low AR levels. Thyroid is also known to express AR in benign and malignant tissue, and AR is known to play a significant role in thyroid cancer development, with higher AR concentration, malignancy rate, and poorer prognosis in males than females[53]. The mechanism, however, by which androgens influence hormonal sensitivity and disease progression in breast and thyroid cancer requires further investigation.

Testicular CIS/ITGCN cells express AR unlike normal adult male germ cells, and may respond directly to androgens after puberty[38], [54]. Paracrine effects of androgens are postulated to be mediated by adult Sertoli cells, which express AR and promote adult germ cell regulation. There is a significantly increased testicular germ cell tumor risk among men with shortened AR CAG repeat length which leads to increased AR transactivation that may be involved in seminoma development or progression of CIS/ITGCN to seminoma[55]. The AR-mediated androgen signals cross-talk with proteins encoded by the Y chromosome (i.e. TSPY), and co-expression of TSPY and AR occurs in testicular germ cell tumors and model cell lines, but not in normal testicular cells[56]. The relationship between USP26 and AR in testicular cancer requires further study.

### Future studies

Study of various types of testicular histologies (SCO, MA, HS, seminoma, nonseminomatous germ cell tumor) for USP26 and AR expression is required. qRT-PCR will further examine the isolated cellular components of testis with the highest expression of USP26 by IF. Preparation of purified Leydig and Sertoli cells should elucidate the localization and expression levels of the USP26 in somatic cells[57], [58]. In addition, human and mice meiotic spreads are being employed in our laboratory. Correlation with hormonal lab values and microdissection TESE outcomes may also be helpful. Several nuclear receptor interaction motifs (L2, L3, F2) were identified in USP26 to result in severely diminished binding to AR when mutated. These warrant further investigation in cell lines and testicular tissue. USP26 is predicted to exhibit deubiquitinating activity according to sequence homology and ubiquitin carboxy-terminal hydrolase (UCH) motif presence. USP26 deubiquitination action and substrate must be verified in humans. Deubiquitinating activity of wild type and mutated variants of USP26 will be compared using deconjugation and co-precipitation assays.

## Conclusion

USP26 mRNA and protein expression and localization have been demonstrated in murine testis and, for the first time, human testis as well as non-testicular benign and malignant tissues. Human USP26, with highest expression in the testis, is localized in testicular Leydig cells with significant nuclear colocalization with androgen receptor. Spermatogonia, primary spermatocytes and Sertoli cells exhibit partial colocalization with androgen receptor. Colocalization of USP26 with AR has been identified in benign and malignant breast and thyroid tissue as well. The role of USP26 in male infertility, hypogonadism, and androgen-dependent cancer requires additional study.

## Supporting Information

Checklist S1 **ARRIVE Guidelines Checklist.**
(DOC)
